# Tai Chi Exercise in Medicine and Health Promotion

**DOI:** 10.1155/2013/298768

**Published:** 2013-11-11

**Authors:** Ching Lan, Steven L. Wolf, William W. N. Tsang

**Affiliations:** ^1^Department of Physical Medicine and Rehabilitation, National Taiwan University Hospital and National Taiwan University, College of Medicine, 7, Chung-Shan South Road, Taipei 100, Taiwan; ^2^Department of Rehabilitation Medicine, Emory University School of Medicine, Atlanta, GA 30322, USA; ^3^Department of Rehabilitation Sciences, The Hong Kong Polytechnic University, Hung Hom, Kowloon, Hong Kong

## 1. Physical Activity and Health

The three major factors that influence our health and longevity are genetics, the environment, and behavior [1]. From the 1980s, the fast development of computer technology has significantly changed human's working environment, behavior, and lifestyle. Physical inactivity and obesity are prevalent worldwide, thus causing a major public health problem. According to the Global Health Observatory Report from the World Health Organization [2], insufficient physical activity is the 4th leading risk factor for mortality. People who are insufficiently physically active have an increased risk of 20% to 30% in all-cause mortality compared to people who engage in moderate intensity physical activity ≧30 minutes for most days of the week. Participation in 150 minutes of moderate physical activity a week or its equivalent is estimated to reduce the risk of ischemic heart disease by approximately 30%, the risk of diabetes by 27%, and the risk of breast and colon cancer by 21%–25% [2].

Traditional exercise training mainly focuses on the younger population and pursuing significant improvement of physical fitness. In the “graying” world, however, new exercise recommendations are needed for the senior population and many “apparently healthy” individuals who may have chronic diseases. Before the mid-1990s, physical activity guidelines recommended vigorous intensity exercise for at least 20 minutes continuously, 3 days a week, with the goal of improving physical fitness and body composition [3]. In 1995, the American College of Sports Medicine (ACSM) and US Centers for Disease Control and Prevention (CDC) [4] issued a landmark report on physical activity and health. The New guidelines recommended that “every adult should accumulate 30 minutes or more of moderate physical activity on most, preferably all, days of the week.” Subsequently, the National Institute of Health [5] and the US Surgeon General [6] issued similar recommendations for physical activity and public health in 1996. These publications called attention to the health-related fitness of regular physical activity, and even moderate exercise intensity that did not meet traditional criteria for improving fitness may significantly improve health [5].

## 2. Principle of Exercise Prescription

A program of regular exercise that includes cardiorespiratory, resistance, flexibility, and neuromotor exercise training beyond activities of daily living to improve and maintain physical fitness and health is essential for most adults [7]. The ACSM recommends that most adults engage in moderate-intensity cardiorespiratory exercise training for ≥30 minutes per day on ≥5 days per week for a total of ≥150 minutes per week [8]. Additionally, adults should also perform resistance exercises for each of the major muscle groups and neuromotor exercise involving balance, agility, and coordination on 2-3 days per week [8]. Crucial to maintaining joint range of movement, completing a series of flexibility exercises for each of the major muscle-tendon groups on ≥2 days per week is also recommended [8]. The exercise program should be modified according to an individual's habitual physical activity, physical function, health status, exercise responses, and stated goals. Behaviorally based exercise interventions and exercise that is pleasant and enjoyable can improve adoption and adherence to prescribed exercise programs. 

## 3. Ancient Wisdom of Exercise

The beneficial health effect of physical exercise has been known dating back to the 5th century BC, when Hippocrates said that “Even when all is known, the care of a man is not yet complete, because eating alone will not keep a man well, he must also take exercise. For food and exercise, while possessing opposite qualities, yet work together to produce health” [9]. In ancient China, the earliest medical book Huangdi Neijing (*Yellow Emperor's Book of Internal Medicine*) described the principle that human harmony with nature was important to prevent disease and was the key to longevity [10]. The ideas in the book have a basis in Chinese Taoist philosophy, and the key to a healthy life is to follow the Tao (e.g., the natural way of the universe). In 1974, the oldest diagram of Taoist exercise (Tao Yin) was excavated from the archeological site of Mawangdui (King Ma's tomb) of the Han Dynasty (206 BC–AD 220) in Changsha, China. Tao Yin is a series of exercises practiced by Taoists to cultivate the internal energy. Tai Chi Chuan is deeply rooted in Taoist philosophy and is well known for its slow and graceful movement. Tai Chi exercise has been practiced for centuries in the East for health promotion and longevity, and it has gained popularity in Western societies recently. During the practice, Tai Chi integrates deep diaphragmatic breathing into continuous body motions to achieve a harmonious balance between body and mind [11]. Tai Chi is performed in a semi squat posture, and the exercise intensity can be easily adjusted by controlling the postural height. The exercise intensity of Tai Chi appears low owing to the slow speed and gentle movements during exercise. The slow exercise speed associated with Tai Chi actually increases the load on the lower limbs in crouching posture, since the poses are held for a comparatively long time. Tai Chi participants with different ages thus can adjust the postural height and duration to achieve similar relative exercise intensity [12].

Tai Chi has several unique characteristics. First, the motions of Tai Chi are slow, harmonious, and relaxing. Second, Tai Chi is performed in a semi squatting posture at extremely slow speed. During the performance, various degrees of concentric and eccentric contraction are demanded for lower extremities [13]. Third, Tai Chi is an exercise with low impact, low velocity, and minimal orthopedic complications [14].

## 4. Tai Chi in Medicine and Health Promotion

In recent years, Tai Chi has become a popular exercise worldwide and research studies are flourishing. Research shows that Tai Chi may be helpful for health-related fitness and can be prescribed safely as a therapeutic exercise for patients with neurological disease, rheumatological disease, orthopedic disease, cardiopulmonary disease, and certain cancer. In this special issue, new evidence is provided by researchers in this field. W. W. N. Tsang and associates measured the reaction time and movement time during performing finger-pointing tasks for older individuals. The findings suggest that Tai Chi may slow down the aging effect on eye-hand coordination tasks involving choices that require more cognitive progressing. There are two studies that evaluated the effect of Tai Chi on breast cancer survivors. S. S. M. Fong and associates applied Tai Chi Qigong to breast cancer survivors and measured the isokinetic peak torques of the shoulder rotator muscles and quality of life. The result showed that greater shoulder muscular strength was significantly associated with better functional well-being in breast cancer survivors with TC Qigong training. In another study, J. L. W. Robins and associates used two stress management interventions (Tai Chi and spiritual growth) for women with breast cancer undergoing adjuvant chemotherapy. The results found that both interventions had insufficient power to overcome the psychosocial or physiological stress experienced during the chemotherapy treatment period. There are three articles that review the clinical applications of Tai Chi. B. Oh and associates reviewed the effect of Qigong on depression, and J. Wang and associates reviewed the effect of Tai Chi on hypertension. C. Lan and associates reviewed the health-promotion effect of Tai Chi and its applications in medicine.

From the perspective of exercise prescription, Tai Chi is a suitable conditioning exercise because the training characteristics fulfill the recommendations of the ACSM regarding exercise to develop and maintain cardiorespiratory function, muscular fitness, neuromotor agility, and flexibility [8]. Moreover, Tai Chi is a low technology approach to conditioning, which can be easily implemented in the community at a minimal cost. Although previous research suggested that Tai Chi has potential benefits for many clinical conditions, large randomized controlled trials with standardized Tai Chi training protocols are needed in future research. 
